# Micro- and nanoplastics-induced stress in plants: uptake, physiological disruption, and toxicity mechanisms

**DOI:** 10.3389/fpls.2026.1772615

**Published:** 2026-03-11

**Authors:** Muhammad Arshad, Gen Li, Irshan Ahmad, Muhammad Shoaib, Tayyub Hussain, Guosheng Chi, Muhammad Asif, Yang Zhou, Huixin Li, Jun Wu, Shixiang Zhang

**Affiliations:** 1 The Sanya Institute of Nanjing Agricultural University, Nanjing Agricultural University, Sanya, China; 2 Soil Ecology Lab, College of Resources and Environmental Sciences, Nanjing Agricultural University, Nanjing, China; 3 Zhejiang Key Laboratory of Crop Germplasm, Department of Agronomy, College of Agriculture and Biotechnology, Zhejiang University, Hangzhou, China; 4 Crop Science Institute, National Agriculture Research Centre, Pakistan Agricultural Research Council, Islamabad, Pakistan; 5 Tobacco Administration Office, Guangze Branch of Nanping Tobacco Company, Nanping, China; 6 Jiangsu Provincial Key Laboratory of Coastal Saline Soil Resources Utilization and Ecological Conservation, Nanjing Agricultural University, Nanjing, China; 7 Zhengzhou Tobacco Research Institute of China National Tobacco Corporation (CNTC), Zhengzhou, China

**Keywords:** crop performance, mechanism, microplastics, nanoplastics, physiological response, phytotoxicity

## Abstract

The persistence and progressive fragmentation of plastic waste into micro- and nanoplastics (M/NPs) represent a significant and escalating threat to agro-ecosystems, adversely impacting a broad spectrum of organisms from soil microbiota to plants, animals, and ultimately human health. Although ecotoxicological studies have documented adverse outcomes, connections between plastic exposure and specific plant physiological endpoints remain underexplored. Here, we synthesize the current evidence on the interaction of plastics particle with plants, especially emphasizing accumulation and distribution of particles in different plant tissues, entry pathways, morphological disruption, and biochemical response. We further devolve into underlying toxicity mechanisms, and synergistic effects with other environmental stressors. Therefore, this review synthesizes current knowledge on the uptake, internal fate, and physiological consequences of M/NPs stress in plants. We also identify key areas for future research, including the development of mechanistic endpoints that directly correlate with crop performance.

## Graphical Abstract

**Figure:**
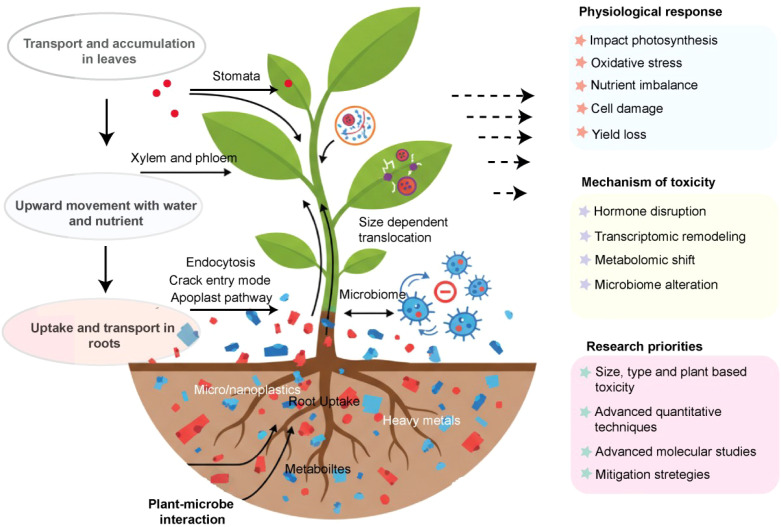
Decoding M/NPs phytotoxicity: uptake, stress mechanisms, and knowledge gaps. This visualization was produced with Perplexity AI (perplexity.ai) and has been critically reviewed and labelled by author.

Decoding M/NPs phytotoxicity: uptake, stress mechanisms, and knowledge gaps. This visualization was produced with Perplexity AI (perplexity.ai) and has been critically reviewed and labelled by author.

## 1 Introduction

Micro/nanoplastics (M/NPs) contamination has emerged as a high-priority environmental issue within soil-plant systems, driven by widespread environmental exposure and the plausible entry of these contaminants into food webs (Browne et al., 2007). Microplastics (MPs) (commonly defined as plastic particles <5 mm) have been detected across marine, terrestrial, and atmospheric compartments, and their occurrence in biota supports concern about trophic transfer and downstream risks (Browne et al., 2007). Plastics are extensively utilized due to their durability, flexibility, and ease of manufacturing (Mendoza et al., 2021). Plastic film is often fragmented into increasingly smaller particles through exposure to solar radiation and mechanical forces, rather than being eliminated from ecosystems (Zhao et al., 2023). Nanoplastics (NPs), characterized by their minute dimensions and high surface-area-to-volume ratio, possess distinct physicochemical and biological behaviors relative to MPs. These properties increase their potential for uptake by living organisms, which can lead to physiological stress in plants and microorganisms, thereby affecting their health and metabolic processes (Koelmans et al., 2022). Therefore, mechanism of uptake and translocation of M/NPs in agroecosystems is essential for both mitigation design and risk evaluation.

Plastic mulching is widely used to conserve soil moisture, regulate temperature, and suppress weeds; however, film aging and fragmentation makes an important pathway for M/NPs accumulation in agricultural crops (Dewi et al., 2024). Plants can internalize these particles through both foliar and root uptake, disrupting standard physicochemical processes, also directly modify the structure and succession of microbial communities, which in turn influences fundamental soil properties (Akhir and Mustapha, 2022). Field surveys reporting high particle loads in agricultural soils relative to adjacent buffer soils underscore the contribution of land use and management practices to M/NPs burden (Lian et al., 2024). For instance, sampled four agricultural soil sites in China, found very high M/NPs concentrations averaging 18,760 particles/kg in agricultural soils. Concentrations were much lower in the forest buffer zone soil, emphasizing the land use effects (Monkul and Özhan, 2021). This justifies the risks associated with plastic mulching practices and the urgency of addressing soil-based contamination in Agricultural crops.

M/NPs enter soils through multiple non-mulch pathways as well, including irrigation inputs, organic amendments, and atmospheric deposition, which together reinforce the need for source-specific control strategies (Koelmans et al., 2022). Early M/NPs research cantered on oceans and other aquatic systems. Studies began to explore areas such as interactions with microorganisms and toxicological effects on biota (Rillig, 2012). However, research on terrestrial ecosystems, particularly regarding the potential impacts of M/NPs on plants, remains in its infancy and represents a minor portion of the overall literature as of now. M/NPs can be internalized by plants via multiple pathways, such as root uptake, foliar absorption, and stomatal entry (Azeem et al., 2021). Following internalization, these particles can translocate and accumulate within various plant structures, including leaves, stems, flowers, and fruits (Liu Y. et al., 2023). This bioaccumulation poses significant concerns regarding the effects of plastic pollution on plant physiology, agricultural productivity, and the safety of the food supply.

**Figure 1:**
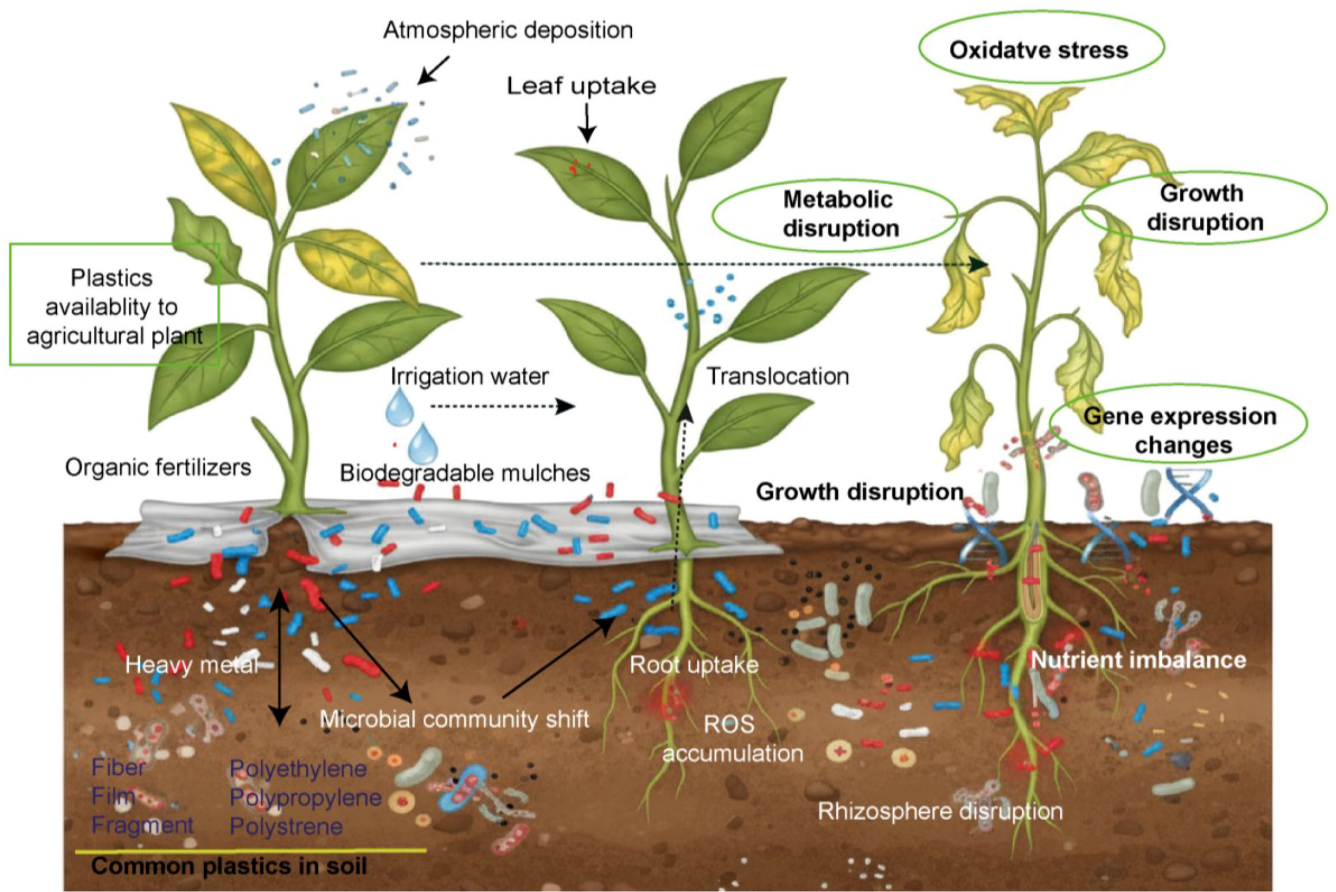
Proposed mechanisms of micro-nanoplastic (M/NP) stress and its cascading effects in the plant-soil system. M/NP uptake triggers a cascade of physiological disruptions, from direct physical interference in the rhizosphere to systemic oxidative stress and signaling dysregulation, ultimately compromising nutrient acquisition, plant growth, and agroecosystem health. This visualization was produced with Perplexity AI (perplexity.ai) and has been critically reviewed and labelled by author.

Recent evidence show that M/NPs exposure is associated with consistent phenotypic and physiological alterations (Guo et al., 2025), oxidative stress (Yu et al., 2021), nutrient imbalance (Liu et al., 2022a), metabolic changes (Liu et al., 2024a), and shifts in gene expression profiles (**Figure 1**) (Arshad et al., 2025a). However, earlier studies focused on gross ecotoxicological endpoints, there remains a relative scarcity of integrated studies that mechanistically link these effects to specific molecular-level disruptions, such as size-dependent cellular internalization pathways, reactive oxygen species signaling cascades, and genotoxic damage. This gap stems from both limited fundamental knowledge of M/NPs behavior and the inherent technical challenges of such investigations. Elucidating the precise mechanisms of M/NPs toxicity in plants is crucial, as it can advanced genetic and molecular breeding approaches aimed at enhancing tolerance. This review synthesizes current literature on the uptake, transport and accumulation of M/NPs within plants. We summarize findings on M/NPs distribution across different plant tissues and detail the specific pathways governing their uptake and internal translocation. The discussion extends to the resultant morphological alterations, analyzes their direct causes, and examines the subsequent adverse effects on plant physiological and biochemical processes along with the underlying molecular mechanism of phytotoxicity. Furthermore, this systematic review briefly addresses the interactive effects of M/NPs with other environmental contaminants.

## 2 Methodology

This review synthesizes current evidence on the phytotoxicity of micro- and nanoplastics (M/NPs), analyzing their uptake pathways, tissue-level accumulation, and subsequent physiological impacts in plants. A systematic search of PubMed, Scopus, and Web of Science (2019-2025) was conducted using key terms including “microplastics,” “nanoplastics,” “phytotoxicity,” “Oxidative stress,” and “plant uptake.” Selected studies were evaluated to determine M/NPs distribution across plant tissues and their effects on growth, oxidative stress, and cellular integrity. Mechanistic insights from metabolic, transcriptomic, and plant-microbiome studies were integrated. Findings were compared to identify dominant trends, clarify current knowledge gaps, and propose targeted research priorities for mitigating M/NPs impacts in agriculture.

## 3 M/NPs in different plant tissue

**Figure 2:**
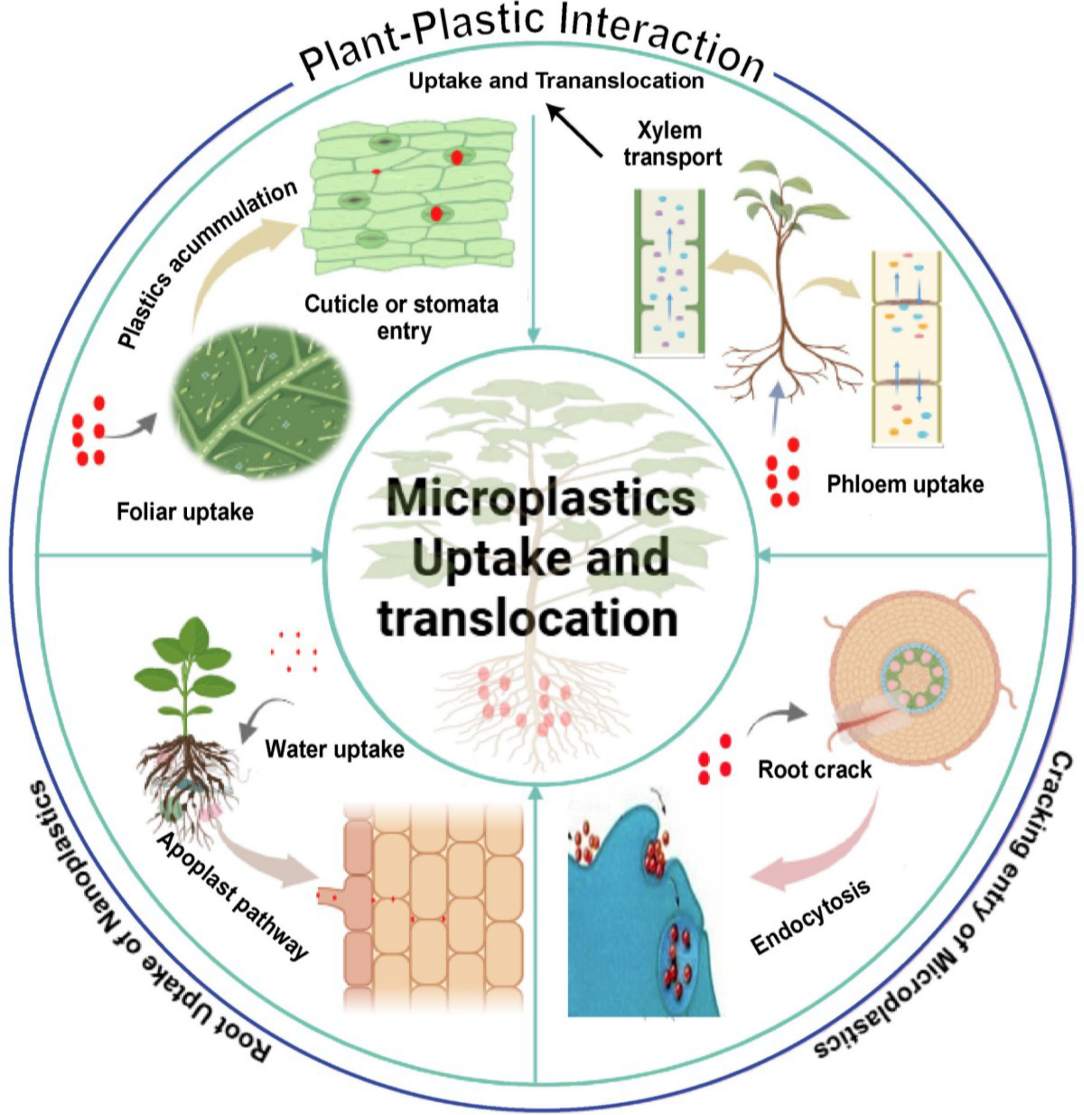
Pathways and tissue-level partitioning of micro-nanoplastic (M/NP) internalization in plants. This illustrates the primary root uptake routes and subsequent systemic translocation, leading to differential particle accumulation in root and leaf tissues, a key determinant of localized phytotoxicity.

Different patterns of transport and bioaccumulation for various M/NPs types are observed across plant species and tissues (**Figure 2**), as summarized in **Table 1**.

**Table 1:** Uptake of plastics in different plant tissues.

Species	T/S	Conc.	Application	d	Observations	Ref.
*Lepidium sativum*	PS-NPs (100 nm)	100 mg/kg	Root	12 days	Nano-plastic presence was confirmed in the root tips, root surface and stele, lateral roots, root hairs, stem vascular bundles, leaf veins and mesophyll, as well as leaf epidermis including stomatal sites	(Sahai et al., 2024)
*Z mays*	PS (NH_2_, COOH)	10mg/L	Foliar	28 d	Both types of plastic accumulate in leaves and further transport to the roots.	(Azeem et al., 2021)
*L. sativa*	PS NPs 93.6 nm	0,1-1mg/L	Foliar	35d	PSNP uptake by plant leaves by stomata and translocation downwards to the plant	(Ilyas et al., 2024)
*Cucumis sativus* L	PS (100–700 nm)	50 mg/kg	Root	65d	PSNPs initially accumulated in the root system before being transported to the aboveground parts of the plant.	(Li Z. et al., 2021)
*Zea mays*	PS (0.2-5 μm)	50 mg/L	Root	10 d	Small PS beads assembled on the cell wall of the xylem, while large PS beads (2.0 μm) were scattered on the cell walls of root xylem	(Li et al., 2023b)
*Phaseolus vulgaris* L., *Sorghum bicolor* (L.) Moench, and *Cucumis sativus* L	PS (0.2-1 μm)	50 mg L^−1^	Root	7d	Uptake and accumulation were higher for small PS beads in *P. vulgaris* and *C. sativus* compared to that in *S. bicolor*, while more large PS beads were accumulated in *C. sativus*.	(Wang et al., 2025)
*Arabidopsis and wheat*	PS 40 nm and 1 μm	0.029 g L*^−^*^1^	Root	12d	Detected in epidermis, cortical tissue, and xylem vessels	(Taylor et al., 2020)
*Lactuca sativa* L.	PE	0.25 mg-1.00 mL^−1^	Root	28 d	Cell rupture and apparent expansion of endoplasmic reticulum vesicles were observed	(Zhu et al., 2022)
*Vicia faba (faba bean)*	100 nm PS beads	100 mg/L	Root	48 h	100 nm PS-MPs can accumulate in *V. faba* root and most probably block cell connections or cell wall pores for transport of nutrients.	(Jiang et al., 2019)
*Triticum aestivum (wheat) and Lactuca sativa (lettuce*)	200 nm and 2.0 mm PS and 200 nm and 2.0 mm polymethylmethacrylate beads (PMMA)	50 mg l^–1^	Root stele, shoots	10 d	particles penetrating the stele of both species using the crack-entry mode at sites of lateral root emergence	(Li et al., 2020)
*Triticum aestivum (wheat) and Zea mays (maize)*	Polypropylene particles (PP) 125 μm	Not Specified	Roots and Foliar	75 d	Accumulation in roots of wheat and stem of maize plant were detected	(Erdem et al., 2023)
Citrus	PSNPs (20 & 50 nm)	Not specified	Root	30 d	PS-NPs are predominantly adhered to the root surface, and no signs of uptake and translocation were recorded in root sections.	(Hussain et al., 2025)
*Arachis hypogaea (peanut), Oryza sativa (rice)*	PS-NPs (80 nm)	250 mg kg^-1^	Root	maturation stage	PS-NP translocation from the root and accumulation in the grains at the maturation stage	(Jiang et al., 2022)
*Solanum lycopersicum (tomato)*	PS-SO3H, PS, F-PS-SO3H (green fluorescently labelled), F-PS-NH2 (green fluorescently labelled), and PS-Eu NPs	Not specified	Foliar	20–30 d	positively charged NPs penetrating more into the leaves and dispersing uniformly within the mesophyll cells.	(Shi et al., 2023)
*Oryza sativa (rice)*	PSNPs (80 nm) and PSMPs (1 μm)	40 mg/L	Root	40 d	Root stele, stem vascular bundles and leaf veins, and mostly aggregated on cell walls and in the intercellular regions	(Liu et al., 2022b)

### 3.1 M/NPs in plant roots, stem and leaves

The root system is a primary site of interaction with M/NPs, functioning as both a critical entry point and a partial filter for environmental contamination (Lin et al., 2024). Initial contact often triggers increased mucilage production, which along with hydrophobic interactions with the cell wall leads to the adsorption of particles onto the root surface, particularly at root caps and hairs (Taylor et al., 2020). The results indicated that plastic particles adsorb and accumulate on the root surfaces, cap cells, and root hairs of both *A. thaliana* and *T. aestivum* (Taylor et al., 2020). While this adsorption can sequester contaminants, it also poses a direct mechanical threat; particles with sharp or rough edges can abrade root tissues, inducing oxidative stress and membrane damage (Li L. et al., 2024). While larger plastic particles cannot be internalized by plant roots, their adsorption onto the root surface can cause mechanical damage, particularly from particles with rough textures or sharp edges (Yu et al., 2024).

Research demonstrated that polystyrene (PS) and polytetrafluoroethylene (PTFE) MPs induce physical abrasion in rice roots. This mechanical damage elevated ROS levels in both roots and leaves, triggering lipid peroxidation and compromising cellular membrane integrity (Dong et al., 2020). More recent evidence indicates that even micrometer-scale plastic particles can enter root systems through fissures formed during lateral root emergence, subsequently translocating to aerial plant components via the transpiration stream (Li et al., 2020). Uptake of M/NPs occurs primarily in root zones with high cellular activity, such as the lateral root junctions and apical meristems (Li et al., 2020). This process is influenced by particle charge; for instance, in *Arabidopsis thaliana*, both positively and negatively charged NPs accumulate, but with distinct distribution patterns. Positively charged particles show limited accumulation in root tips, whereas negatively charged variants are frequently localized in the apoplast and xylem vessels (Sun et al., 2020). The resultant accumulation within root tissues can obstruct cell wall pores or disrupt intercellular connections, thereby impeding the transport and absorption of water and nutrients, which ultimately inhibits plant growth (Rajput et al., 2025).

The upward transport of M/NPs within plants is primarily driven by transpirational pull, with higher transpiration rates generally increasing particle mobilization. Once particles enter the xylem vessels, they are carried passively with the transpiration stream toward aerial tissues (Habibi et al., 2025). Consequently, exposure to elevated concentrations of plastics is consistently linked to inhibited shoot development, including reduced stem elongation and diminished aboveground biomass (Nigina and Nair, 2024). This phytotoxicity is likely mechanistically linked to the particles’ presence in the vascular system. M/NPs, particularly through aggregation or due to their inherent size, can physically obstruct xylem conduits, impairing hydraulic conductance and the distribution of water and nutrients (Chaudhary et al., 2025). This vascular dysfunction represents a direct pathway through which stem-transported M/NPs compromise plant growth and productivity.

Leaves are a major sink for M/NPs, receiving particles via multiple routes: systemic translocation from the roots, direct atmospheric deposition, and adhesion to the leaf surface (Lin et al., 2024). Once on the foliage, particles can become trapped in microstructures like trichomes and the cuticle or, if sufficiently small, enter through stomatal openings (Srećkov et al., 2025). Electrostatic interactions further modulate surface adherence, with positively charged particles showing greater affinity for typically anionic leaf surfaces. Foliar accumulation of M/NPs directly impairs photosynthesis. Exposure correlates with decreased chlorophyll content, reduced photosynthetic rate, and altered stomatal conductance (Gao et al., 2021; Pehlivan et al., 2026). The primary mechanism involves the physical obstruction of stomata by aggregated or larger particles, which blocks gas exchange. Furthermore, M/NPs can disrupt the expression of photosynthesis-related genes, indicating a multifaceted attack on the photosynthetic apparatus (Pehlivan et al., 2026). Consequently, the foliar uptake and internal translocation of M/NPs can substantially disrupt plant physiological processes, ultimately impaired growth and reduced productivity.

### 3.2 Mechanisms of uptake and transport

Current research points to several key pathways, including apoplastic transport, a “crack-entry” mode at lateral root junctions, stomatal penetration, and possibly endocytotic processes in certain contexts (**Figure 2**).

#### 3.2.1 Endocytosis

The internalization of M/NPs cannot occur via membrane channels or transporters due to their size and polymeric nature. While endocytosis has been proposed as an effective uptake route, its biological relevance for particles exceeding one micron is precluded by fundamental biophysical constraints (Hua and Wang, 2022). Definitive evidence for endocytotic entry is limited to studies using small-sized plastic particles (<100 nm) in isolated cell cultures. For example, 40 nm polystyrene beads can be internalized by cultured tobacco BY-2 cells, but this capacity ceases for particles approaching 100 nm (Yu et al., 2024). Critically, this upper size limit (100 nm) falls far below the recognized threshold for microplastics (>1000 nm), aligning with the known diameter of endocytic vesicles (70–180 nm), which cannot accommodate micron-scale particles (Yu et al., 2024). Furthermore, key demonstrations of uptake, such as the entry of particles into sycamore cell vacuoles, required the use of protoplasts cells with their walls artificially removed. This model eliminates the primary physical barrier to particle entry in plants and therefore does not represent a physiologically relevant pathway (Pal et al., 2025). Consequently, while endocytosis may be a valid mechanism for nano-sized plastics entry in simplified systems, there is no empirical evidence supporting its operation for large-sized particles in intact plants.

#### 3.2.2 Apoplastic transport

Plant root tips are highly active and sensitive regions. Their cells produce large amounts of mucus and secretions when stimulated. Before plastic particles can enter the root, most M/NPs are initially captured by this sticky root surface layer, which acts as a primary filter and may prevent easy internalization at the very tip (Schwab et al., 2020). Despite the mucilage barrier, a small fraction of M/NPs can be taken up with water. They travel via the apoplastic pathway moving through the gaps between cell walls without entering the cells themselves. This extracellular route allows particles to move from the root epidermis toward the central vascular tissue (Pal et al., 2025). Key biophysical barriers exist along this route, most notably the Casparian strip in the endodermis, which blocks passive apoplastic diffusion into the vascular cylinder (Sun et al., 2020). A study on lettuce (*Lactuca sativa*) seedlings confirmed the apoplastic transport of submicron plastic beads within root tissues. While 0.2 µm beads were observed moving through intercellular spaces along cell walls, this finding suggests that nano-scale particles can more readily access this extracellular pathway (Li et al., 2019). Similarly, Studies in *Arabidopsis* found that negatively charged NP reached the vascular bundle via the apoplast, while positively charged ones did not. Positives charges likely cause particles to stick to negatively charged root surfaces or mucilage (Driouich et al., 2021). Even when particles enter the xylem, transport to shoots is limited.

#### 3.2.3 Crack-entry mode

This process bypasses the size-exclusion limits imposed by cell wall pores and plasmodesmata, which are generally on the order of 5–20 nm and thus too small to permit passive diffusion of micron-scale particles (Sun et al., 2020). Instead, plastics exploit natural, transient fissures that form at sites of lateral root emergence and within the actively dividing tissues of the root apical meristem, where the Casparian strip is not yet fully developed. Studies in wheat and lettuce have demonstrated that submicrometer- and micrometer-scale PS and polymethyl methacrylate particles can use these cracks to enter the stele and subsequently accumulate within xylem (Li et al., 2020). Confocal microscopy of wheat cross-sections confirmed that M/NPs entering via this route are primarily confined to the root and stem vasculature. These particles were detected inside xylem conduits within two hours, translocated via the transpiration stream, and had migrated to cortical tissues within twelve hours (Li et al., 2020). This pathway now recognized as a primary route for the systemic transport of M/NPs, effectively circumventing the traditional apoplastic barriers like the endodermal Casparian strip (Yin, 2024).

#### 3.2.4 Stomatal entry

Recent evidence indicates that small-sized M/NPs can be taken up by leaves, primarily through stomatal openings or potentially via cuticular penetration (Sun et al., 2021). Once internalized, these particles can enter the phloem vasculature. Driven by source-sink pressure gradients, they are then translocated downward-from leaves to stems, and to roots (Wang et al., 2020). This phloem-mediated pathway mirrors the transport dynamics observed for other engineered nanoparticles (e.g., metal oxides) but is defined by the sub-micron scale required for stomatal entry and phloem loading.

Crucially, this foliar-to-root pathway has been demonstrated only for nanoparticles. The size exclusion of stomatal apertures and the loading constraints of phloem sieve tubes preclude the uptake and systemic phloem transport of larger MPs particle through this route (Yin, 2024). Consequently, while representing a significant vector for nanoparticle contamination in crops, this mechanism is not considered a relevant uptake pathway for M/NPs in plants.

### 3.3 Size, surface charges and chemical properties of M/NPs

The size of M/NPs is a primary determinant of their capacity for plant uptake and internal translocation. Accumulating evidence indicates that smaller particles particularly those within the NPs range are more readily absorbed by plant roots and subsequently mobilized through vascular tissues. For example, when cucumber plants were exposed to PS-NPs of varying diameters (100, 300, 500, and 700 nm), only the smallest (100 nm) particles were efficiently internalized and translocated to aerial tissues, leading to measurable reductions in growth and physiological performance (Li Z. et al., 2021). The limited uptake of larger particles is attributed to their inability to penetrate the rigid cellulosic cell wall and the pectin-rich matrix, which can reach several micrometres in thickness, thereby acting as a strong size-exclusion barrier (Lin et al., 2025). Comparable observations have been reported in other species. In *Vicia faba*, exposure to PS-NPs of varying sizes revealed, via laser confocal scanning microscopy, that 100 nm NP accumulated extensively in root tissues, potentially obstructing plasmodesmata and apoplastic pores that facilitate nutrient transport. In contrast, larger particles (5 μm) were seldom detected within root tips, highlighting the critical role of particle size in determining cellular accessibility (Jiang et al., 2019).

Further evidence supporting the influence of size on M/NP transport is provided by tissue distribution studies. A study demonstrated that 1 μm particles could enter carrot roots and localize within intercellular spaces, though with low frequency and no subsequent transport to leaves (Dong et al., 2021). In contrast, particles within the 50-150 nm range were consistently observed both within intercellular layers and leaf tissues, suggesting that nanosized plastics can traverse the apoplastic continuum a key route for systemic transport (Dong et al., 2021). Similarly, a study reported that exposure of *Lepidium sativum* to progressively smaller plastic particles (50, 500, and 4 800 nm) resulted in a marked decrease in germination rate, likely due to nano-sized particles obstructing seed-coat pores and impeding water uptake (Bosker et al., 2019). The observed reduction in germination rate is likely attributable to the physical obstruction of seed coat pores by plastic particles, which impedes water imbibition and consequently delays germination. These findings underscore that particle size is a decisive factor governing M/NPs absorption, internalization, and physiological impact in plants. The effective translocation of M/NPs requires particles to traverse multiple structural and biochemical barriers, the properties of which vary substantially among species, developmental stages, and environmental contexts. Consequently, the threshold size enabling plant uptake and transport is not fixed but dynamically modulated by plant anatomy and growth conditions.

The mechanical properties of M/NPs, particularly their elasticity, are essential for their uptake and transport within plants. The polymer’s mechanical strength enables M/NPs to potentially enter plant tissues via a crack-entry mode at points like the root apex. Since the mechanical strength of common M/NPs (e.g., ~2.1 GPa for PS beads) is generally lower than that of the plant cell wall, they can be compressed and deformed during internalization (Zhao et al., 2024). For instance, studies show smaller PS-NPs (100–300 nm) undergo significant morphological changes and edge blurring during root-to-shoot transport, while larger particles (500–700 nm) largely retain their original shape (Adhikari, 2024). This deformation may result from: i) mechanical shear stresses during upward vascular transport exceeding the particle’s cross-resistance, or ii) chemical interactions with plant exudates (e.g., organic acids, enzymes) in the vasculature that degrade the polymer matrix.

The surface charge of M/NPs critically influences their interaction with plant tissues and subsequent uptake. Plant cell walls and membranes typically carry a net negative charge, creating an electrostatic barrier (Sun et al., 2020). Consequently, positively charged M/NPs exhibit strong adsorption to these surfaces, competing with cations for binding sites, which often limits their internalization and systemic transport. For example, positively charged NPs accumulate more at root tips but show reduced entry into the xylem compared to their negatively charged counterparts (Sun et al., 2021). Accurately detecting M/NPs within plant tissues is hindered by several interconnected challenges. The diverse physicochemical properties of M/NPs complicate method standardization, with less universally accepted protocols for their efficient extraction from complex plant matrices rich in interfering organic compounds (He et al., 2022). Sample contamination and the inherently low concentrations of M/NPs present significant hurdles for precise quantification, pushing the limits of current analytical techniques like microscopy and spectroscopy.

## 4 Inhibitory impacts of M/NPs on plant growth: root and shoot responses

Once inside M/NPs have an inhibiting influence on plant growth, as evidenced by plainly visible morphological and physiological alterations. Research shows that both M/NPs particles have a significant impact on plant growth and development across a wide range of species and experimental practices. Recent studies have showed that M/NPs accumulate in plant roots, altering different parameters (**Table 2**) linked to root growth and characteristics, such as root length, relative root elongation, root activity, and fresh and dry weight, among others (Tang, 2020). A study reported that exposure to ≥1.5% biodegradable MPs (Bio-MPs) (100µm) significantly reduced root and shoot biomass in common bean. This study found that Bio-MPs at concentrations of 1.5%, 2.0%, and 2.5% w/w decreased root biomass to 3.80 ± 0.43 g and 3.75 ± 0.16 g, compared to 4.31 ± 0.49 g in the control treatment (Meng, 2021). Another study observed that both low-density polyethylene (LDPE-MPs) and Bio-MPs (20nm) at concentrations of 1.5%, 2.0%, and 2.5% w/w significantly inhibited root and shoot biomass in *Phaseolus vulgaris* (Meng et al., 2023). The study also noted that Bio-NPs treatments and ≥1.0% LDPE-MPs showed significantly higher numbers of specific root nodules. Soybean growth was significantly inhibited by the presence of 0.1% polylactic acid microplastics (PLA) (20-60 µm) (Lian et al., 2022). Interestingly, both M-NPs appear to exert comparable detrimental effects on plant growth, highlighting the broad and persistent impact of plastic pollutants across different particle sizes.

**Table 2:** Micro/nanoplastics disrupt the plant growth and development effect plant yield.

Type	Plant	Conc. (mg/kg)	Response observed on plant	Ref
PS (5 μm)	*Solanum tuberosum* L.	100 - 500	After 100d exposure Morphological parameters, including seed germination rate, shoot and root lengths, leaf area, and fresh and dry biomass of plants, got reduced significantly with the increase in MP concentration.	(Qaiser et al., 2025)
PS (5–7 µm)	*Triticum aestivum* L.	0%, 1%, 3%, and 5% (*w*/*w*)	Although all shapes of PS significantly reduced morphological growth traits, PS in powder shape was the microplastic that reduced plant height (by 58–60%), fresh biomass (by 54-55%) and dry biomass (by 61–62%) the most, especially at the 3% and 5% concentrations compared with 0% PS	(Riaz et al., 2025)
PS (5 μm)	*Zea mays* L	5.0%, and 10.0%	45 d exposure results showed that PS contamination significantly reduced maize growth, with the highest PS concentration (10.0%) leading to reduction in root dry weight from 2.86 to 2.09 g, chlorophyll content from 32 to 14, and plant height from 73.67 to 52 cm	(Rassaei, 2025)
PS (73 ± 3 nm)	*Glycine max* L.	0.075	A 14-day exposure resulted in a significant reduction in both shoot and root length	(Dini et al., 2025)
PS (20–30 nm)	Glycine max L.	0.125, 0.25, 0.5	A 7-day exposure significantly reduced both the fresh and dry weight of shoots and roots, along with a decrease in shoot length	(Surgun-Acar, 2022)
PS (19 ± 0.16 nm)	*Oryza sativa* L.	0.01	A 16-day exposure resulted in an increase in the number of lateral roots	(Zhou et al., 2021)
PS (19 ± 0.16 nm)	*Oryza sativa* L.	0.05	A 7-day exposure led to a decrease in root length	(Dong et al., 2020)
PS (10 µm)	*Oryza sativa* L.	0.1 and 0.2	Seven days of exposure resulted in a decrease in both root length and root biomass	(Dong et al., 2020)
PS (5.64 ± 0.07 µm)	*Hordeum vulgare* L.	2	A 7-day exposure resulted in a reduction in the number of rootlets	(Lian et al., 2020)
PS (52.48 ± 20.93 μm)	*Lycopersicon esculentum* L	0.01-1	2 days exposure reduced root length, root dry weight and root fresh weight, also reduced shoot fresh weight	(Shi et al., 2022)
PP (20nm,100um)	*Nicotiana tabacum*	100 and 1000	A 55-day exposure significantly reduced root length, root dry weight, and root fresh weight. Additionally, shoot fresh weight was also reduced, with the effects worsening at higher concentrations	(Arshad et al., 2025a)
PP (20nm)	*Zae mays*	500	A 48-day exposure significantly reduced both root and shoot fresh biomass	
PP (20nm,100um)	*Nicotiana tabacum*	100 and 1000	A 45-day exposure significantly reduced root length, root dry weight, and root fresh weight. Additionally, shoot fresh weight was reduced, with the effects worsening at higher concentrations	(Arshad et al., 2025b)
PP (40–50 µm)	*Cucurbita pepo* L	0.02-0.2	A 16-day exposure reduced shoot dry weight, shoot fresh weight, root dry weight, and root fresh weight, with the effects becoming more pronounced at higher concentrations	(Colzi et al., 2022a)
PP (< 500 μm)	*Solanum lycopersicum* var. *cerasiforme*	0.1	A 9-day exposure significantly reduced root elongation	(Shorobi et al., 2023)
PE (40–50 µm)	*Cucurbita pepo* L	0.02, 0.1, 0.2	A 16-day exposure significantly affected shoot dry weight, while no effect was observed on root or shoot fresh weight	(Colzi et al., 2022b)
PE (20–50 µm)	*Glycine max* L	0.1 and 1	A 49-day exposure increased shoot length at a 0.1 concentration, while no significant effect was observed at higher concentrations	(Lian et al., 2022)
PE (23 µm)	*Lactuca sativa* L. var. *ramosa Hort*	2.5, 5, 1	Exposure for 14 and 28 days significantly reduced plant growth, including leaf fresh weight, leaf dry weight, plant height, and number of leaves	(Gao MinLing et al., 2019)
PE (4–10 mm)	*Triticum aestivum* L.	1	Exposure for 60 and 120 days reduced plant biomass	(Qi et al., 2020)
PE (125 µm)	*Triticum aestivum* L.	10, 20	A 14-day exposure reduced both root and shoot biomass, while higher concentrations increased root biomass	(Liu et al., 2025)
PE (75.37 ± 17.55 μm)	*Lycopersicon esculentum* L	0.01, 0.1, 0.5, 1	A 2-day exposure significantly reduced germination, root length, and root weight, with higher concentrations having a more pronounced effect	(Lindert et al., 2025)
PE (20nm,100um)	*Nicotiana tabacum*	100, 1000	Both sizes at both concentrations negatively influenced plant growth, significantly decreasing plant height, as well as shoot and root length	(Arshad et al., 2025b)
PVC (40–50 µm)	*Cucurbita pepo* L	0.02-0.2	A 16-day exposure reduced shoot dry weight, shoot fresh weight, root dry weight, and root fresh weight, with the effects being more pronounced at higher concentrations	(Santini et al., [[NoYear]])
PVC (125 µm)	*Triticum aestivum* L.	1, 5, 10 and 20	A 14-day exposure negatively affected both shoot and root biomass, while higher concentrations showed a positive effect on root biomass	(Zang et al., 2020)
PVC (125 µm)	*Triticum aestivum* L.	1, 5	54-day exposure increased both shoot and root biomass at both concentrations, but negatively affected the shoot-to-root ratio	(Liu Z. et al., 2022)
PVC (15 µm)	*Zea mays* L.	0.1, 1,10	30-day exposure negatively affected plant height and shoot biomass at higher concentrations	(Zhang et al., 2023)
PET (40–50 µm)	*Cucurbita pepo* L	0.02-0.2	16-day exposure significantly affected root fresh weight and shoot dry weight, with more pronounced effects at higher concentrations	(Colzi et al., 2022b)
PET (< 0.45 µm)	*Cicer arietinum* L	0.25, 0.75, 1	2-day exposure affected the germination rate	(Mondal et al., 2022)
PET (5–60 µm)	*Lepidium sativum*	0.002	6-day exposure increased shoot height and leaf number, but decreased the germination rate	(Pignattelli et al., 2021)
PBAT (11.11–161.81 µm)	*Arabidopsis thaliana*	0.2	A 49-day exposure decreased total leaf area, dry weight, and the number of fruits	(Liu J. et al., 2022)
PBAT (0.25–5 mm)	*Glycine max* L.	1	28-day exposure reduced flowering and root length	(Yu Y. et al., 2023)
PBAT	*Zea mays* L.	1, 2, 5, 10	28-day exposure reduced flowering, root length, and root harvest yield	(Yu Y. et al., 2023)
PBAT (250–1000 µm)	*Phaseolus vulgaris* L	(0.05 and 0.1)	18-day exposure increased root biomass at low concentrations	(Meng et al., 2021)

Furthermore, plastics of varying sizes induce distinct forms of stress in plants, with the underlying mechanisms differing based on particle size. Larger MPs stick to the outer root surface, inducing physical damage, hindering plant growth, and disrupting nutrient absorption (Dan et al., 2025). In contrast, smaller NPs are uptake by plant roots zone via cellular endocytosis, accumulating within the plant and further impeding its development (Dan et al., 2025). Further research is crucial to fully understand the physiological impacts of these two distinct forms of microplastic-induced damage on plant health.

## 5 M/NPs-induced oxidative stress and cellular damage in plants

**Figure 3:**
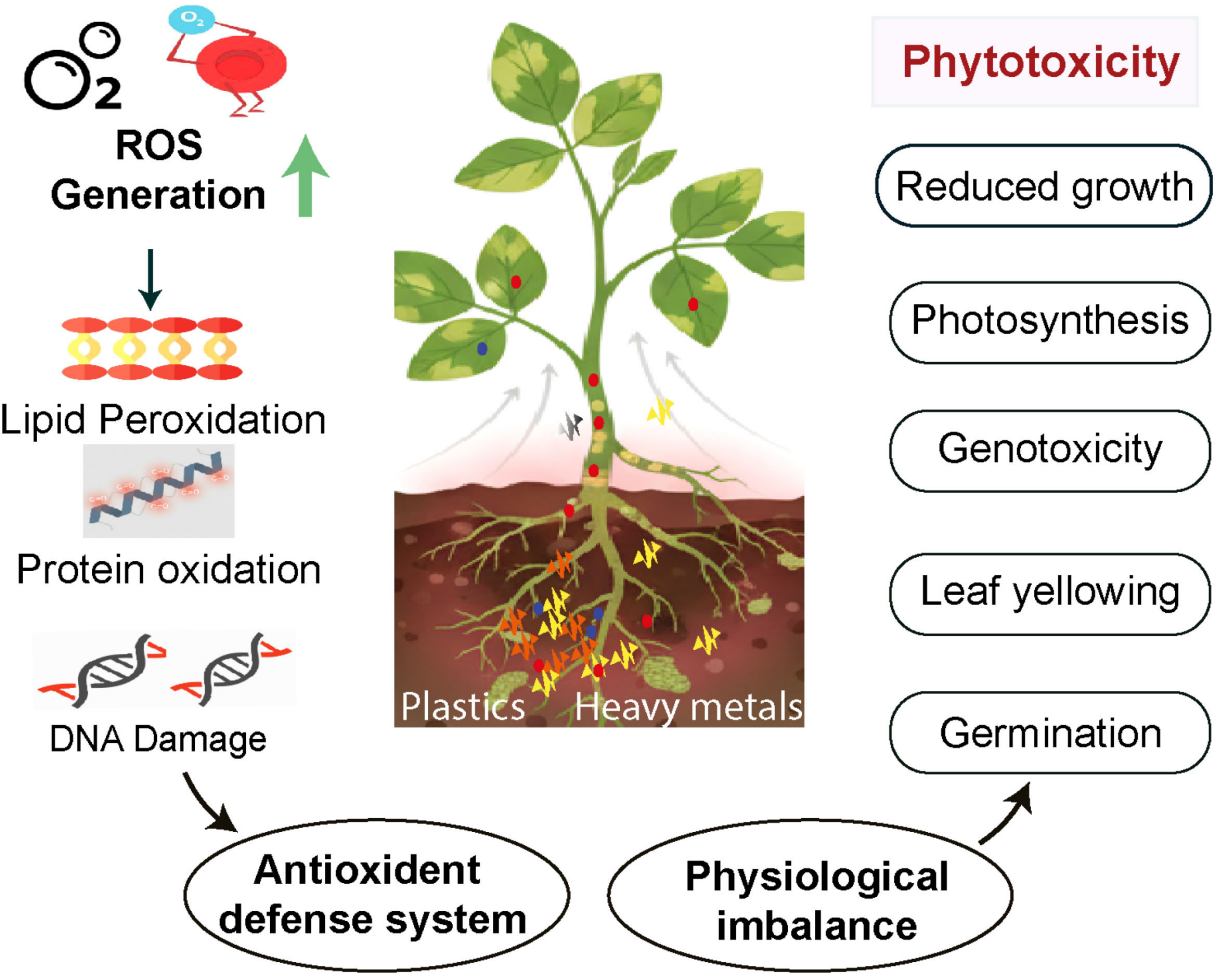
The toxicity of M/NPs to plants extends beyond visible effects, with their mechanism involving the passive regulation of physiological and biochemical processes through the induction of oxidative stress.

Under external stress, plants accumulate higher levels of reactive oxygen species (ROS), such as hydrogen peroxide, superoxide anion, hydroxyl radicals, and singlet oxygen, which can cause irreversible damage (Basumatary et al., 2026). M/NPs similarly provoke oxidative stress via excessive ROS production, with H_2_O_2_ and O_2_^–^ serving as key, readily detectable biomarkers (**Figure 3**). This imbalance manifests as lipid peroxidation quantified by malondialdehyde (MDA) accumulation compromising membrane integrity, alongside protein carbonylation and DNA strand breaks (Hu et al., 2024). Physically, M/NPs can interfere with root systems, altering soil physicochemical properties and consequently disrupting nutrient uptake and water absorption in plants. This disturbance in nutrient and water balance can itself elicit stress responses that promote ROS generation (Pang et al., 2023). For instance, Exposure to 75 mg L^–1^ of 73 ± 3 nm PS-NPs elevated H_2_O_2_ and O_2_^–^ levels in soybean roots, concomitant with upregulated catalase (CAT) activity and downregulated peroxidase (POD) activity (Qiu et al., 2023). Moreover, M/NPs can act as carriers for other pollutants, such as heavy metals and antibiotics, resulting in combined toxicity that amplifies oxidative stress in plants (Li et al., 2022). In a study on wheat seedlings (*Triticum aestivum* L.), simultaneous exposure to PS particles and arsenate resulted in significantly heightened phytotoxicity compared to individual stressors, demonstrating a clear synergistic interaction between M/NPs and co-occurring contaminants (Cao et al., 2024). This combined stress exacerbated both mechanical damage and oxidative stress in plant cells, leading to increased accumulation of ROS and elevated lipid peroxidation in root tissues. In response, the activities of key antioxidant enzymes, including SOD and POD, were markedly upregulated (Cao et al., 2024).

Plants counter ROS via enzymatic antioxidants SOD, CAT, and POD (Jiang et al., 2024). SOD catalyzes the conversion of O_2_^–^ into H_2_O_2_, which is subsequently detoxified by CAT and POD into water and oxygen (Ma et al., 2023). However, exposure to M/NPs can significantly impair the functioning of these enzymes. For instance, in adzuki bean (*Vigna angularis*) and rice, different types and concentrations of PS, polyvinyl chloride (PVC) and Polyethylene particles negatively affected antioxidant enzyme activity, leading to enhanced oxidative damage (Liang et al., 2024). Recent work in cherry radish and Chinese cabbage confirms non-monotonic responses, with low-to-mid PS-MP doses (10–50 mg kg^-1^) elevating MDA via root-surface ROS generation (Chen et al., 2025).

This unquenched ROS erodes plasma membrane fluidity via peroxidation, triggers genotoxicity, and curtails photosynthesis, yielding chlorosis, stunted growth, germination failure, and yield deficits across crops (Kadac-Czapska et al., 2024). Such damaging effects of M/NPs have been reported across various plant species, including soybean (Li et al., 2023a), maize (*Zea mays* L.) (Zhang et al., 2023) and blackgram (Sahasa et al., 2023). In soybean, M/NPs disrupted the glyoxalase system a key detoxification pathway resulting in impaired growth (Khan et al., 2024). In maize seedlings, shoot biomass exhibited a significant dose-dependent reduction in response to exposure to PVC. As a defense mechanism against this stress, the antioxidant enzyme activities of superoxide dismutase and catalase were markedly elevated in the seedling leaves (Zhang et al., 2023). Recent studies indicate exposure to M/NPs robustly triggers oxidative damage in plant tissues (**Table 3**). Therefore, it is essential to establish threshold levels for different M/NPs types, concentrations, and particle sizes.

**Table 3:** Effects of micro/nanoplastic exposure on plant oxidative stress markers and antioxidant enzyme activity.

Type	Crop	Size	Time	Tissues	Ref
PVC	Glycine max L.	13 μm	75 d	Increased MDA content by 21.8-97.9%, while decreased net photosynthesis rate and caused severe oxidative stress	(Li et al., 2023a)
PE	Nicotiana tobacum	20 nm, 100 µm	55 d	Increased Peroxidation and hydrogen peroxide compared to control	(Arshad et al., 2025a)
PVC	Zea mays	15 µm	30 d	To defense the impact of PVC-MPs, the superoxide dismutase and catalase activities in seedlings leaf were stimulated	(Zhang et al., 2023)
PS	Triticum aestivum L	87 nm	21 d	Significant increase in lipid peroxidatidtion was observed	(Lian et al., 2020)
PS	Onion	50 nm	72 h	Increase in lipid peroxidation and hydrogen peroxide accumulation was observed	(Giorgetti et al., 2020)
PS	Arabidopsis thiana	71 nm	7 w	Increase hydrogen peroxide and superoxide anion level modified gene expression	(Sun et al., 2020)
PS	Rice	19 nm	16 d	decrease the hydrogen peroxide and TBARS level while increase the APX and SOD activity in root	(Zhou et al., 2021)
PS	sweet pepper (*Capsicum annuum* Linn.)	1.2 μm		The size of MPs significantly impacts their accumulation characteristics in C. annuum roots, leading to variations in toxic mechanisms, including oxidative stress and damage.	(He et al., 2024)
PP	Nicotiana Tabacum	100 μm	48 d	The induction of severe oxidative stress, evidenced elevation in malondialdehyde (MDA) levels, was directly associated with a significant reduction in the net photosynthetic rate.	(Arshad et al., 2025b)
PE	Tobacco	20nm	48 d	Signifcant increase in Peroxidation and hydrogen peroxide cause the oxidative stress	(Arshad et al., 2025a)

## 6 Underlying mechanisms of phytotoxicity

### 6.1 Plant metabolism under plastics stress

Plant metabolic reprogramming is a sensitive indicator of M/NPs phytotoxicity because it integrates upstream stress perception with downstream growth and yield outcomes (Lv et al., 2023). Recent studies suggest that M/NPs exposure can affect photosynthesis (Maes et al., 2025), phytohormone homeostasis (Zhu et al., 2025), and core carbon-nitrogen metabolism (Hu et al., 2025), with glycolysis (Cheng et al., 2025) and related pathways frequently reported as responsive nodes. Metabolomics strengthens mechanistic inference by linking phenotype-level inhibition to pathway-level perturbations and candidate biomarkers of M/NPs stress (Fu et al., 2025).

Photosynthesis is repeatedly identified as a key functional target of M/NPs exposure, with proposed drivers including reduced light capture, chloroplast structural injury, and oxidative stress-mediated damage to photosynthetic machinery (Wu et al., 2019). A recent meta-analysis discovered that M/NPs exposure lowers photosynthesis by 7.05-12.12% in terrestrial crops, marine algae, and freshwater algae. This study also indicated that present ambient M/NPs levels could cause yearly yield losses of 109.73-360.87 million metric tons in major crop output and a 0.31-7.24% decrease in aquatic primary productivity (Zhu et al., 2025). It have been shown to impede soybean growth and development by reducing the synthesis of L-tryptophan, a critical precursor for auxin production, as well as salicylic acid 2-O-β-glucoside in soybean leaves under PS-M/NPs exposure (Qiu et al., 2023). In a similar fashion, PS-M/NPs inhibit rice biomass accumulation by blocking the biosynthesis of lignin and jasmonic acid (Zhou et al., 2021). Furthermore, soybean metabolomics studies have highlighted that PS-M/NPs disrupt key metabolic processes, affecting nitrogen, carbon, ascorbate, and aldarate metabolism (Shoaib et al., [[NoYear]]). Notably, PS-M/NPs have also been shown to alter the carbon cycle in barley (Li S. et al., 2021). These metabolic shifts offer valuable insights into the toxicological impact of M/NPs, as alterations in plant metabolism serve as a sensitive indicator of environmental stress. By examining the metabolic responses of plants to M/NPs exposure, changes in key metabolites can be used to evaluate toxicity levels. While research has been conducted on the effects of M/NPs on specific metabolic pathways, a comprehensive and systematic understanding remains elusive. Further studies are needed to delineate the full spectrum of metabolic disruptions caused by M/NPs stress, paving the way for more accurate assessments of their ecological risks.

### 6.2 Genotoxic response of plastic stress

The detrimental effects of M/NPs on animals have been clearly documented. Similarly, they can cause cytotoxicity and genotoxicity in plants (Chen et al., 2022). M/NPs exposure changes the expression of a wide range of genes in plant roots and leaves, including stress responses, metabolism, hormone signaling, cell cycle regulation, and non-coding RNAs (Golubska et al., 2025). Micronucleus (MN) frequency is critical for measuring oxidative damage in plants (Hadi and Stopper, 2025). M/NPs treatment at 100 mg/L inhibited mitosis and increased MN frequency in V. faba, leading to oxidative damage (Wang et al., 2025). Alterations in plant normal biochemical or physiological processes in plants are often driven by changes in gene regulatory networks, which play a crucial role in mediating plant responses to environmental stressors (Leong et al., 2025). These networks control gene expression levels, which in turn determine the production of proteins that regulate key processes such as plant growth, development, and stress responses (Soleymani et al., 2025). Through the fine-tuning of gene expression, plants can adapt to environmental changes by modulating the activity of enzymes, hormones, and other molecular players essential for survival. This intricate regulation underscores the complexity of plant responses to both biotic and abiotic stresses, where even subtle shifts in gene expression can have profound effects on overall plant health and productivity (Soleymani et al., 2025). Currently, our understanding of plant responses to M/NPs stress is primarily based on transcriptome data (Xu et al., 2025). However, identifying genes related to M/NPs exposure remains a challenge, as it requires the creation of transgenic lines and the verification of downstream protein functions, which is a time-consuming process. To advance our knowledge of the molecular toxicity of M/NPs in plants, future research should prioritize identifying and characterizing MPs-related functional genes. In wheat (Triticum aestivum L.), exposure to PS-MPs has been shown to disrupt carbon metabolism and plant hormone signaling, as well as alter the mitogen-activated protein kinase (MAPK) signaling pathway. These changes suggest that microplastics may play a role in the complex cascade of plant-pathogen interactions (Khosrovyan et al., 2025). Although transcriptome analysis provides a solid foundation for investigating plant-M/NPs interactions, this approach is still in its early stages.

### 6.3 Plastics alter the normal rhizosphere soil microbial community

**Figure 4:**
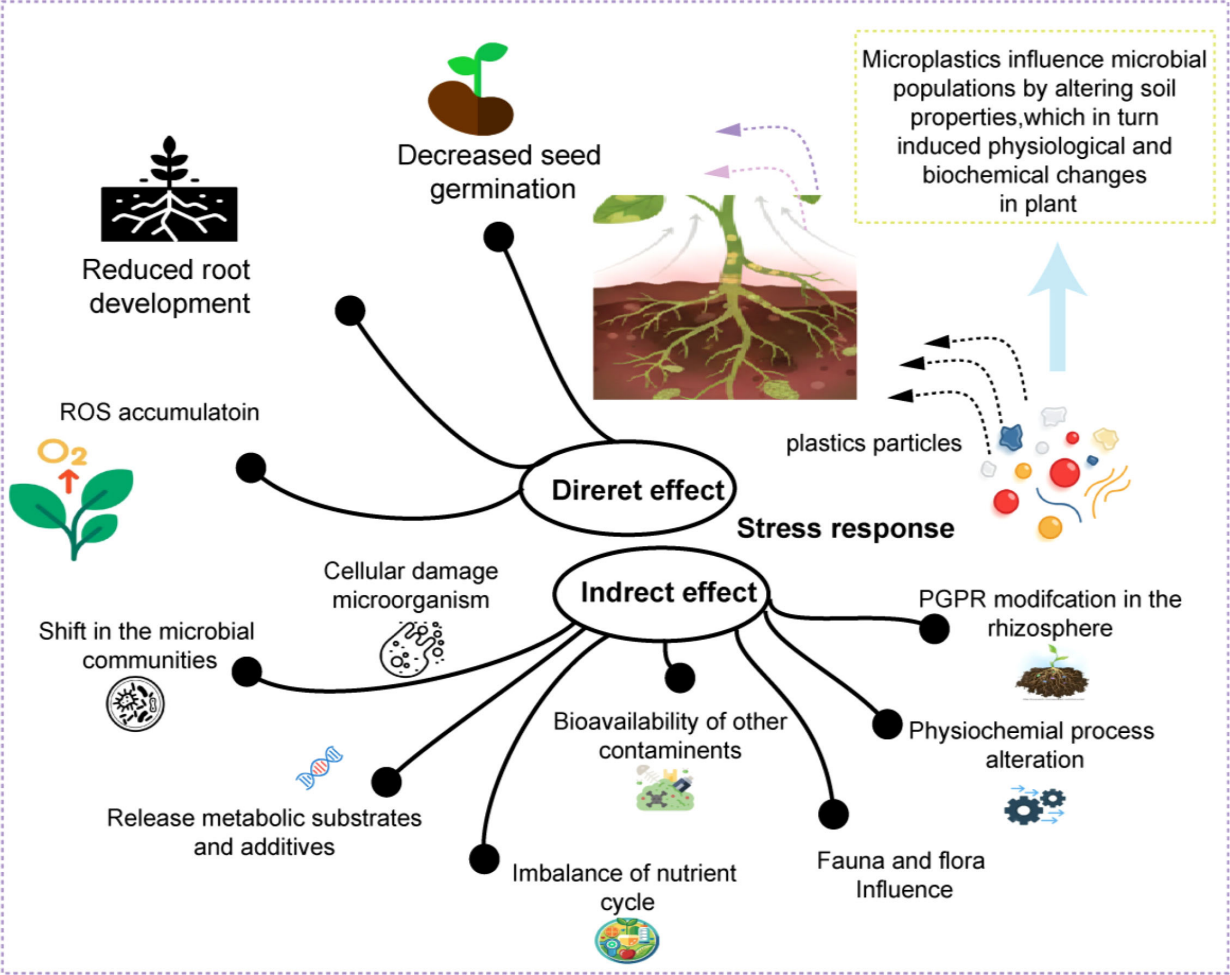
Plastics disrupt plant health via rhizosphere community dysbiosis. M/NP-induced soil property alteration to shifts in microbial diversity and function, which subsequently impair nutrient cycling and symbiotic signaling, ultimately driving plant physiological stress.

M/NPs have a significant impact on rhizosphere microbial communities and overall ecosystem functioning, influencing both directly and indirectly (**Figure 4**). These particles can disrupt microbial diversity and alter microbial interactions within the rhizosphere, which in turn affects nutrient cycling, soil health, and plant growth. Directly, M/NPs can physically obstruct microbial habitats, while indirectly, they may introduce chemical contaminants or change the soil’s physical properties, leading to shifts in microbial populations. These disruptions can have cascading effects on ecosystem services, highlighting the need for further research into the ecological implications of M/NPs pollution in soil environments (Hidalgo-Ruz et al., 2012). Microbial interactions are key drivers of plant growth and development, and the dynamics of plant-microbe interactions have been extensively studied (Ren et al., 2022). M/NPs influence microbial populations by altering soil properties, which in turn induces physiological and biochemical changes in plants. The impact of M/NPs on plant-microbe interactions highlight their toxic effects (Ren et al., 2022). A study examining soybean plants exposed to PE and polylactic acid (PLA) MPs for 49 days revealed significant shifts in microbial biodiversity, particularly in the rhizosphere. The bacterial communities in the rhizosphere showed heightened sensitivity to PLA-MPs, leading to more pronounced alterations compared to polyethylene exposure (Guo et al., 2025). Despite growing concern, there has been limited research on how M/NPs affect plant growth and development through alterations in microbial communities, and the underlying mechanisms remain poorly understood.

### 6.4 Interaction with other environmental stressors

**Figure 5:**
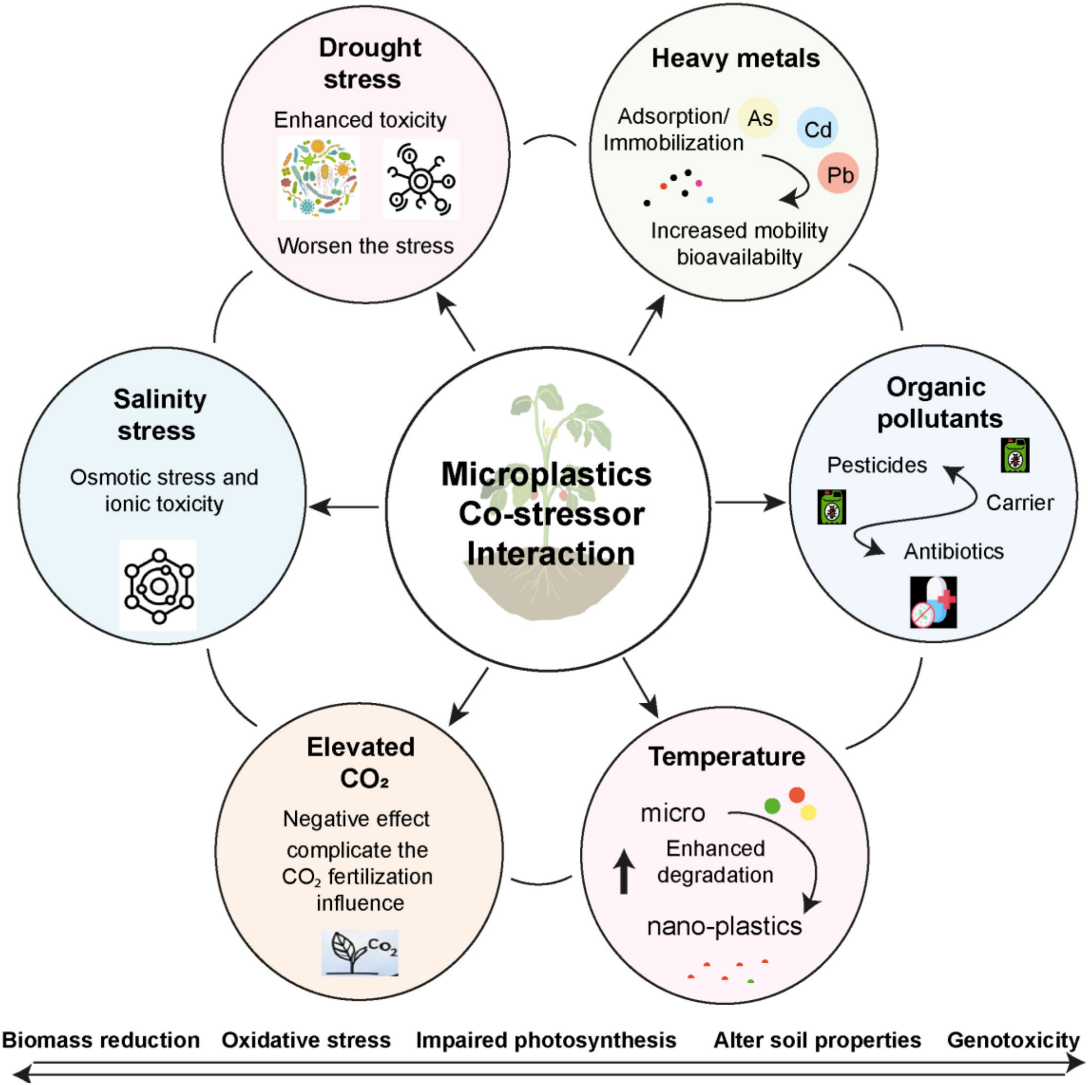
Mechanistic framework for the combined toxicity of plastics and co-stressors in plants. This model illustrates how microplastics act as a vector and stress amplifier, interacting with abiotic and biotic stressors to produce synergistic, antagonistic, or additive phytotoxicity via shared pathways like oxidative burst and metabolic interference.

M/NPs are ubiquitous environmental pollutants that interact with various abiotic stressors, significantly affecting plant growth and physiology (**Figure 5**). These interactions often lead to more severe impacts on plants than either stressor alone (Shah and Aslam, 2025). One prominent documented environmental stressor interacting with microplastics is heavy metal contamination (Pu et al., 2026). Studies have consistently shown that MPs can act as carriers for heavy metals, altering their mobility, bioavailability, and subsequent uptake by plants (Liu et al., 2021). For instance, the combined exposure of PE-MPs and cadmium (Cd) has been observed to reduce the photosynthetic performance of maize (*Zea mays* L.) (Li Y. et al., 2023). Specifically, PE-M/NPs can lead to increased Cd accumulation in plants, impacting chloroplast function and reducing the efficiency of photosystem II, which is crucial for photosynthesis (Li Y. et al., 2023). In sorghum (*Pennisetum glaucum* L.), the co-exposure of PVC-MPs and mercury chloride (HgCl_2_) exacerbated negative impacts on plant growth, biomass, photosynthetic pigments, and gas exchange characteristics, while increasing oxidative stress (Al-Huqail et al., 2024). The presence of PENPs also affects the phytoremediation efficiency of heavy metals like Cd, Pb, and Zn by plants such as *Solanum photeinocarpum* and *Lantana camara*, by altering the accumulation of these metals in plant tissues (Gudeta et al., 2023). This highlights a complex interplay where M/NPs can either promote or hinder heavy metal uptake depending on the specific metal, MP type, and plant species (Yu Q. et al., 2023). The interaction between M/NPs and heavy metals primarily involves sorption and desorption processes, influenced by M/NPs type, aging, concentration, size, and soil properties (Liu et al., 2024b). Similarly, M/NPs in agricultural soils can exacerbate the adverse effects of drought on plants. For instance, low-density polyethylene (LDPE-MPs), when combined with drought stress (20% plant available water), significantly impacted lettuce (*Lactuca sativa*) physiology, growth, and root development more severely than either stressor alone (Barone et al., 1001). This combined stress can lead to reduced plant biomass, alterations in physiological parameters, and impaired root architecture, crucial for water uptake. Conversely, some studies indicate that drought can alleviate the negative effects of M/NPs on soil micro-food web complexity and stability, suggesting a nuanced interaction that varies with specific conditions (Liu M. et al., 2023). However, the general consensus is that M/NPs interfere with soil hydraulic properties and water retention, intensifying water scarcity for plants under drought conditions.

Soil salinity presents a dual challenge, often compounded by M/NPs presence. High salinity leads to osmotic stress and ionic toxicity, affecting water absorption and cellular metabolism in plants (Qiu et al., 2024). While direct studies specifically on M/NPs -salinity co-stress from the provided literature are limited, the underlying mechanisms of M/NPs-induced physiological stress such as oxidative stress, nutrient uptake interference, and root damage would likely be amplified under saline conditions (Mészáros et al., 2023). For example, M/NPs can influence root development, which is critical for a plant’s ability to cope with osmotic stress by exploring soil for water (Barone et al., 1001). The combined impacts of M/NPs and other pollutants extend to organic contaminants and antibiotics. Due to their adsorptive capabilities, can serve as vectors for organic pollutants and antibiotics, increasing their persistence and potentially their toxicity in the soil (Xie et al., 2025). For example, combined contamination of PE-MPs and antibiotics (norfloxacin/doxycycline) significantly altered the composition of microbial communities and metabolism in wheat and maize rhizosphere soil, with consequences for plant growth (Zhang et al., 2024). Research has shown that M/NPs can affect plant responses to organic pollutants such as ibuprofen, simazine, sertraline, and amoxicillin (Martín Fernández et al., 2023). Invasive plant species can also be affected by microplastic presence. The increase in alien plant invasions poses a significant threat to global biodiversity, and M/NPs, as an environmental stressor, can impact the interactions between invasive and native species (Meng et al., 2024). Studies have shown that soil M/NPs can alter invasive plant community performance and the dominance of species like *Amaranthus palmeri* (Meng et al., 2024). The promoting effects of soil MPs on alien plant invasion can also depend on the microplastic shape and concentration (Li G. et al., 2024). Similarly, Plants exposed to combined high CO_2_ and M/NPs exhibited higher biomass than those under either stressor alone (Jian et al., 2023). This suggests that MPs may interact with environmental factors, such as CO_2_ levels, influencing plant responses in unexpected ways. Research on barley has demonstrated that PS-MPs reduce plant resilience to low-temperature stress, while also amplifying the toxicity of zinc oxide nanoparticles (Wang et al., 2022). The magnitude of these impacts depends on factors such as MP concentration, size, shape, polymer type, and the specific plant species.

## 7 Conclusion and prospect

M/NPs have been shown to enter plants through multiple routes, including both root and foliar uptake. Once absorbed, these particles can be redistributed throughout the plant, accumulating in roots, stems, and leaves (Shi et al., 2024). M/NPs entry occurs via several distinct mechanisms’ endocytosis, apoplastic transport, crack-entry modes at root junctions, and in some cases, penetration through stomatal openings on leaf surfaces. This uptake and root-surface attachment of M/NPs disrupt key physiological processes, leading to phenotypic alterations and diminished crop yield. Among the various parameters affecting these processes, particle size is the dominant factor determining M/NPs bioavailability and systemic translocation. While the effects of particle size are relatively well documented, the influence of M/NPs shape has received comparatively little attention. Experimental evidence indicates that fibrous M/NPs are less likely to cross root barriers or move upward within vascular tissues than spherical microbeads, yet fibers often induce stronger toxicological responses in plants (Lozano and Rillig, 2020; Agarwal et al., 2023). This discrepancy underscores a key research gap, as M/NPs in soils primarily exist as fibers and fragments rather than as idealized microspheres commonly used in laboratory studies (Khalid et al., 2020). Future work should prioritize understanding how particle morphology influences both the uptake pathways and the subsequent physiological impacts on plants under field-realistic conditions. Also, many studies utilize MNPs concentrations that are considerably higher or less than those typically found in agricultural soils or natural waters. This discrepancy limits the direct applicability of laboratory findings to real-world scenarios (Kokalj et al., 2024).

The detection of M/NPs in various agricultural crops both root vegetables such as carrots, radishes, and potatoes, and aboveground crops such as wheat, rice, and lettuce highlights their potential to enter the human food chain (Lian et al., 2021; Zhou et al., 2021). Once ingested, these particles may persist and bioaccumulate due to their nanoscale dimensions and chemical persistence. Additionally, M/NPs carry the chemical additives such as phthalates and bisphenol A, have been found in brain (Nihart et al., 2025) and placentas (Ragusa et al., 2021) which disrupt the immune and reproductive systems (Burgos-Aceves et al., 2021), though the exact route of entry remains unclear. Studies further indicate that exposure to M/NPs induces oxidative stress and inflammatory responses in biological systems, emphasizing their potential to threaten human health (Yu et al., 2024).

A comprehensive understanding of M/NPs behavior in plants requires further investigation across several points. Developing rapid, quantitative detection methodologies will allow real-time tracking of particle movement within plant tissues. Comparative studies should also focus on the kinetics and transport mechanisms across species and M/NPs types, encompassing both composition and morphology. Elucidating the terminal accumulation points of M/NPs within edible tissues will be crucial for accurate assessment of agricultural contamination and food safety risks. Finally, many experimental studies utilize MP concentrations that are considerably higher than those typically found in agricultural soils or natural waters. This discrepancy limits the direct applicability of laboratory findings to real-world scenarios.
